# Supplementary material for: Preparation and Boron Removal Performance of Glycidol Modified PANI Nanorods: An Optimization Study Based on Response Surface Methodology
Source: Polymers (Basel). 2023 Jan 15;15(2):459. doi: 10.3390/polym15020459 (PMC9864539; doi:10.3390/polym15020459)
Supplement: Supplementary file 1 [file polymers-15-00459-s001.zip › polymers-2089332-supplementary.pdf]

Article

Preparation and boron removal performance of glycidol modified PANI nanorods: an optimization study based on response surface methodology

Yunlong Le, Yunshan Guan *, XiaoYing Ma and Weidong Zhang *

College of Chemical Engineering, Qinghai University, Xining 810016, China

* Correspondence: qh-gys@163.com (Y.G.); weidzhang1208@126.com (W.Z.); Tel.: +86-18997298841 (W.Z.); Tel.: +86-13186052872 (Y.G.)

Table S1. Low- and high-level values for the independent variables

Factors	Symbol	Unit	Code level	
			Low level	High level
Adsorption time	A	h	6	10
Boric acid concentration	B	mg/L	1200	1400
pH	C	—	9	11
Response	Adsorption capacity	mmol/g		

Table S2. Parameters of kinetic model fitting for PANI-OH adsorbed boron

Models and parameters	Pseudo-first-order kinetics model			Pseudo-second order kinetic model		
	$\ln(q_e - q_t) = \ln q_e - k_1 t$			$\frac{t}{q_t} = \frac{1}{k_2 \times q_e^2} + \frac{t}{q_e}$		
	k_1	q_e	R^2	k_2	q_e	R^2
value	0.2848	0.2116	0.9950	1.044	0.2844	0.9919

Table S3. Fitting parameters of internal diffusion model of adsorbate to adsorbent PANI-OH

Model parameter	Intraparticle diffusion model		
	$q = kt^{0.5} + c$		
	k	c	R^2
value	0.0621	0.01836	0.94931

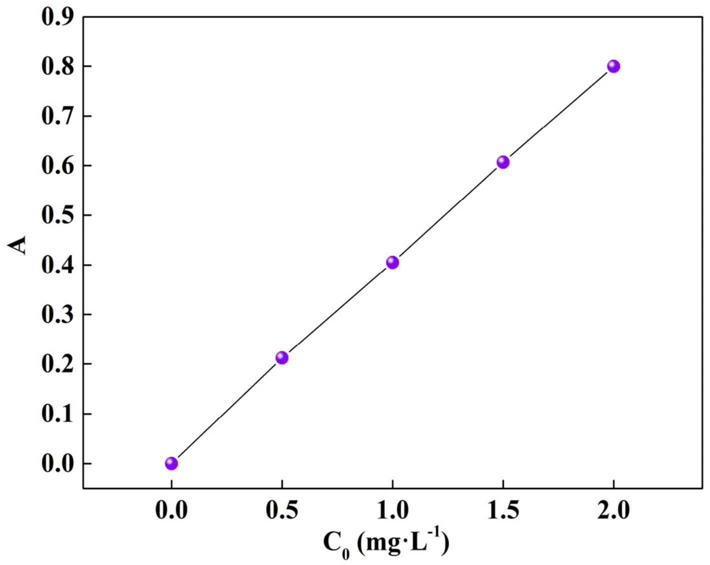

Figure S1. Standard curve of boron concentration

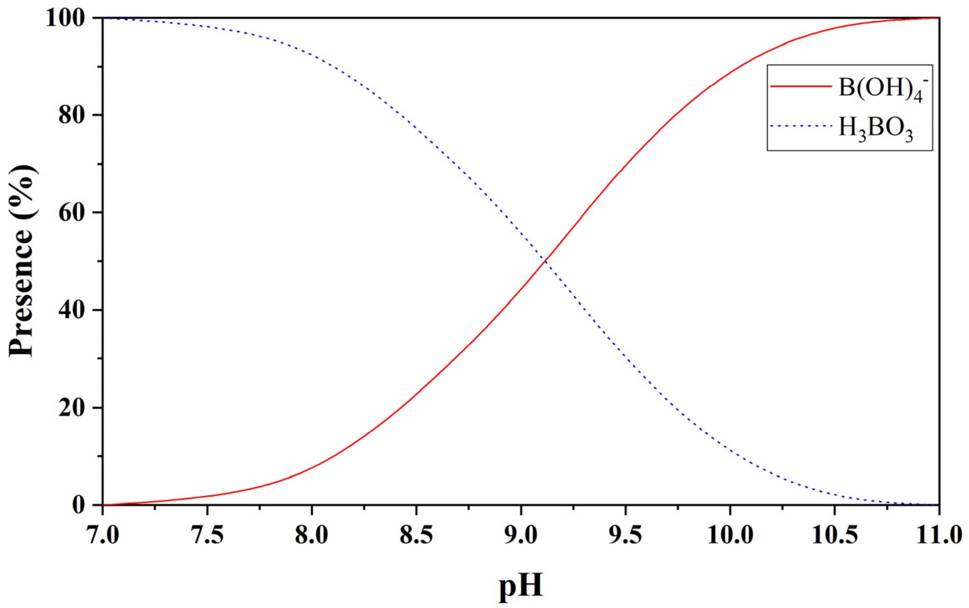

Figure S2. Fraction of boron in the aqueous solution at different pH values.
